# Digital financial and banking competition network: Evidence from China

**DOI:** 10.3389/fpsyg.2022.1104120

**Published:** 2023-01-30

**Authors:** Kaiwei Jia, Ying He, Mohammad Mohsin

**Affiliations:** ^1^School of Business Administration, Liaoning Technical University, Huludao, China; ^2^School of Business Administration, Liaoning Technical University, Fuxin, Liaoning, China; ^3^Department of Management Sciences, Virtual University, Faisalabad, Punjab, Pakistan

**Keywords:** digital finance, banking competition, credit, state-owned, homogenization problems

## Abstract

The rapid development of digital finance resulted in fierce competition in the banking industry. The study used Bank-corporate credit data to measure interbank competition based on social network model, and we transformed the regional digital finance index into bank-level digital finance index using each bank’s registry and license information. Furthermore, we employed QAP (quadratic assignment procedure) to empirically test the effects of digital finance on the competitive structure among banks. Based on which, we verified its heterogeneity and investigated the mechanisms through which the digital finance affected the banking competition structure. The study finds that, digital finance reshapes the banking competition structure, and intensifies the inter-bank involution while increasing the evolution. The large nation-owned banks have been in central position in the banking network system, with stronger competitiveness and higher strength of digital finance development. For large banks, digital financial development has no significant impact on inter-bank co-opetition and is only more significantly correlated with banking weighted competitive networks. For small and medium-sized banks, digital finance has a significant impact on both co-opetition and competitive pressure. Meanwhile, digital finance also led to the increasing homogeneous competition. In addition, compared with large nation-owned banks, the competitiveness of small and medium-sized joint-equity commercial banks and urban commercial banks are more vulnerable to digital finance and resulting in homogenization problems. Mechanism analysis showed that (1) digital finance promotes the overall competitiveness of the banking industry by improving the inclusiveness of financial services, which expands the service scope (scale effect); (2) digital finance promotes the competition by improving the pricing ability, risk identification ability and finally the capital allocation ability of banks (Pricing effect). The above findings provide new ideas for the governance of banking competition and the realization of a new pattern of economic development.

## 1. Introduction

Banks have played a pivotal role in the financial system and economic growth of the countries. Since China’s economic reform and opening up for international firms, the number of banks has gradually grown during the rapid development of the economy. With the establishment of the National Financial Stability Bureau and the National Anti-Monopoly Bureau, the government has paid enough attention to banking competition, but there are still many problems which need to be addressed. X. H. Zhang^①^ suggested in the Global Financial Forum 2019 that one of the existing problems of China’s banks is that they are big in size but not strong. There are number of reasons for this, for instance, banking industry in China has been controlled by government for the long time that resulted in insufficient competition. Secondly it is observed that individual banks still have monopolistic behavior. Therefore, increasing the banking competition to serve the real economy in China is still an important task.

The concept of digital finance was first introduced in 2002 in China. With the impetus of the internet and digital technology, digital finance as a new business model has brought great changes to traditional financial institutions. As digital finance is still in its initial development stage, there are still controversies about the effects of digital finance on the development or competition of the banks, and its intrinsic mechanism remains unexplored (Xie and Zou, 2012; Wu and Wang, 2021). In order to advance research in this field, new breakthroughs in research methodology should be found. The emergence of social networks not only provides a new perspective for the research on this topic, but also enables us to rethink the issue in an environment dominated by indirect financing, capturing the interaction between banks and banks, the interaction between banks and enterprises, and the interaction between enterprises and enterprises. So, it is interesting to know whether banking competition network among banks will discover the role of digital finance? Or whether digital finance improves banking competition? If it does, does digital finance change large banks more or change small and medium-sized banks more? Why and how digital finance improve banking competition?

The study finds that, digital finance reshapes the banking competition structure, and intensifies the inter-bank involution while increasing the evolution. The large nation-owned banks have been in central position in the banking network system, with stronger competitiveness and higher strength of digital finance development. At the same time, the effect of digital finance accelerated the competition among banks, but also led to the increasing of homogeneous competition. In addition, compared with large nation-owned banks, the competitiveness of small and medium-sized joint-equity commercial banks and urban commercial banks are more vulnerable to digital finance and resulting in homogenization problems. Mechanism analysis showed that (1) digital finance mainly promotes the overall competitiveness of the banking industry by improving the inclusiveness of financial services, which expands the service scope (scale effect); (2) digital finance promotes the competition mainly by improving the pricing ability, risk identification ability and finally the capital allocation ability of banks (Pricing effect). The above findings provide new ideas for the governance of banking competition and the realization of a new pattern of economic development.

For this research, complex network science offers fresh viewpoints and resources that enable an in-depth investigation of relationships among banks, companies based on credit data. The marginal contributions mainly include three aspects: (1) building competition networks (weighted competitive networks and co-opetition networks) using credit data to evaluate competition in the banking sector. (2) Using the QAP (quadratic assignment procedure) method to examine the effects of digital finance on banking competition. (3) Enriching mechanism of digital finance affecting bank competition.

The paper is structured as follows. The second part is a literature review; the third part explains the research design, explaining the construction of banking competition network, illustrating the transformation of regional digital finance index into bank-level digital finance index; the fourth part is the empirical analysis and the fifth part is the mechanism analysis. Finally, the sixth part concludes the study.

## 2. Literature review

The relationship between digital finance and competition in the banks has been widely discussed. Especially, the impact of digital finance on banking competition has received more attention from domestic and international academics. Most studies suggest that digital finance has reshaped the business model and competitive structure of the traditional banking industry (Xie and Zou, 2012; Dapp, 2014; Jagtiani and Lemieux, 2018; Fu and Wang, 2021; Wu and Wang, 2021; Bejar et al., 2022).

The first view is that digital finance has promoted competition among banks (Xie and Zou, 2012; Wu and Wang, 2021; Bejar et al., 2022). Wu and Wang (2021) from the perspective of individual banks, found that the development of digital finance increased the degree of competition and competitive pressure as (Xie and Zou, 2012; Feng and Guo, 2019). Tambunlertchai et al. (2021) from the regional perspective, showed that digital finance increases the degree of competition in the banking sector in regions. Feng and Guo (2019), and Xie and Zou (2012) all concluded that the development of digital finance has increased the level of competition and competitive pressure in the banking industry.

The second viewpoint argues that digital finance reduces the degree of competition among banks. According to Dapp (2014), digital finance has contributed a shift from the single competition to co-opetition among banks, while alliances in cooperation reduced potential competition.

In addition, a few scholars have also argued that digital finance has not affected inter-bank competition (Jagtiani and Lemieux, 2018; Fu and Wang, 2021). Jagtiani and Lemieux (2018) argued that digital finance only makes up for the supply gap in traditional services and has not affected the core business of commercial banks. According to Fu and Wang (2021), the development of digital finance will increase inter-bank cooperation rather than promote bank competition.

It is obvious that academics have widely differing perspectives on the relationship between digital finance and banking competition, which requires further verification and clarification. The mixed results about digital finance may be due to different ways to measure digital finance and competition. To explore the relationship between digital finance and banking competition, scientific measurement of digital finance and banking competition is indispensable. There are four main approaches to measuring digital finance in academia. First, text mining techniques are used to measure the digital finance index of individual banks, which is based on the number of keywords searched in search engine entries such as Google. However, this method is susceptible to subjective factors and the accuracy of the data is insufficient (Zhang and Liu, 2017). The second method is to use the ratio of digital transaction amount to bank capital to characterize the development of digital finance in banks (Feng and Guo, 2019). Although this ratio can measure the impact of digital finance on banks to some extent, it is still inadequate because digital transactions are only one component of digital finance. Third, the FinTech regulatory sandbox is used as a proxy variable for this indicator (Samuel and Derrick, 2020). This approach is still up for argument because it is currently in the pilot phase of the FinTech regulatory sandbox in China. Fourth, the Digital Inclusive Finance Development Index developed by Peking University was used to assess the level of development of digital finance in each province and city (Guo et al., 2020). However, the index does not describe the digital finance development of individual banks and is better suited for regional studies. To fill the gaps in the existing research methodology and to keep a tight grip on the scope of digital finance, we transformed the regional level digital finance index into bank-level digital finance index using the information on banks’ domiciles and licenses. Regarding the measures of banking competition, existing articles mainly focus on two categories. First, Measurement based on reduced form model or statistical methods, including the Herfindahl index, market concentration, and amount of bank loans (McPherson, 1983; Rhoades, 1993; Berger et al., 2004). Second, structural models based on bank operating indicators, such as the Lerner index (Lerner, 1934; Liu and Cuevas, 2021). According to Beck et al. (2011), it is impossible to analyze banking competitiveness without including the covariate behavior of banks and interactions between banks and companies. So, it is crucial to portray banking competition from the perspective of inter-bank and bank-company relationships and to analyze the impact of digital finance on competition. For this research, complex network science offers fresh viewpoints and resources that enable an in-depth investigation of relationships among banks, companies based on credit data. Therefore, from a network perspective, whether and how will digital finance alter banking competition? The marginal contributions mainly include three aspects: (1) building competition networks (weighted competitive networks and co-opetition networks) using credit data to evaluate competition in the banking sector. (2) Using the QAP method to examine the effects of digital finance on banking competition. (3) Enriching mechanism of digital finance affecting bank competition.

## 3. Research design

### 3.1. Banking competition measurements

Social networks are derived from the development of network science, the core of which is to analyze the relationship between individuals and analyze the structural condition of the whole society or system from the relationship. Nodes serve as hubs in the network, connected edges serve as ties to establish relationships, degree is an important indicator of node attributes, and centrality provides the basis for identifying important nodes. To this end, the following section will further explore the bank-to-bank relationship using the social network model to portray the inter-bank competition variables in the form of a network.

**Figure 1:**
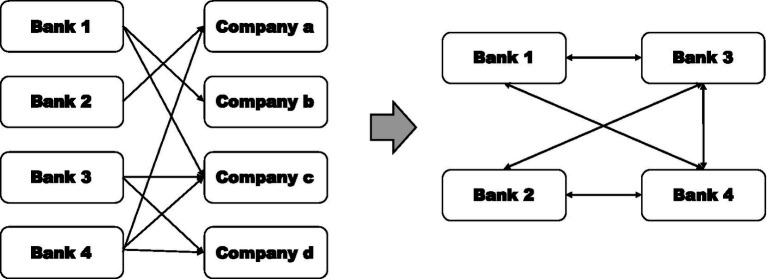
Network relationship conversion diagram.

In order to better explore the competition structure among banks, and to research the internal relationship between banks and companies or banks and banks, this paper measures and portrays the competition relationship among banks from two dimensions. Firstly, from the perspective of inter-bank relationship, the construction and visualization results of the competitive network are used to analyze the loan competition and cooperation relationship between banks, set as CCNet (Co-opetition Network). Specifically, we use the credit data of banks and companies to organize and clean the raw data. In order to reduce node redundancy and increase the visibility of network results, this paper converts the non-standardized two-mode network data into one-mode network data, that is the relationship data between banks and enterprises is converted into the relationship data between banks and banks, and the specific relationship conversion schematic is shown in Figure 1.

Secondly, a weighted competitive network is established by considering the competitive strength of banks. In order to better measure the competitiveness of banks on the basis of the coopetition network, this paper introduces the concept of market commonality pioneered by McPherson (1983), which is a theoretical model that can not only reflect the competitive relationship between nodes, but also measure the competitive pressure of individual banks. Therefore, this paper estimates a WNet based on the market commonality model, using the value of market commonality as the network weight. The specific market commonality model is:


(1) $$CWNet_{ij}=\overset{k}{\underset{a=1}{\sum }}\frac{\ln_{ia}}{\ln_{i}}\times \frac{\ln_{ja}}{\ln a}$$

Where $Ln_{ia}$ denotes the loan amount of bank i to firm a , $Ln_{i}$ denotes the total amount of funds lent by bank i , $Ln_{ja}$ denotes the loan amount of bank j to firm a , and $Ln_{a}$ denotes the amount of loan received by firm a .

### 3.2. Banking digital finance measurement

Financial institutions are the core of digital finance development, and the combination of new technology such as big data, cloud computing, artificial intelligence and traditional financial business has created the digital era as well as digital finance. Regional digital finance development is closely related to local and surrounding financial institutions. On the one hand, regional digital finance development affects financial institutions, and financial institutions also make great contributions to the local digital finance construction.

Based on the research of He and Hu (2022), this paper uses information on the licensing and registration locations of banks as weights to transform the provincial digital finance index to bank-level digital finance index. A model was developed specifically to measure the index through the distribution of each bank in China’s 31 provinces, autonomous region and municipality. Specifically, using the registered locations of each bank’s head office, branches, and sub-branches as the digital financial development weight of the bank in the provincial area, we measure the value of digital financial development of each bank in China. The specific measurement model is as follows:

(2) $$Df_{i}=\overset{31}{\underset{j=1}{\sum }}\frac{n_{ij}\times Le_{ij}}{{\left(\sum n_{i}\times Le_{i}\right)}_{j}}\times RDf_{j}$$

Where $Df_{i}$ denotes the degree of digital financial development of each bank, denotes the number of branches of bank i in province j, denotes the level of banking institutions (the head office level is assigned as 3, branch level is assigned as 2, and the sub-branch level is assigned as 1), and is the financial development index of each province in China measured by the Digital Finance Research Institute of Peking University.

### 3.3. Empirical model construction

#### 3.3.1. Quadratic assignment procedure

The QAP was first proposed by Krackhardt (1987) for studying the relationships between matrices. It is a method to obtain matrix-to-matrix correlation coefficients by comparing the similarity between two matrices. Specifically, the procedure converts each matrix into a long vector, estimates the coefficients between the two long vectors, then, subsequently permutes a matrix row and its corresponding column randomly to obtain the correlation coefficient distribution, and compare it with the first obtained correlation coefficient after 100 or even 1000 of iterations to determine the relationship between the two matrices. This method can not only avoid the problem of multi-collinearity between the explanatory variables and the control variables (Liu, 2007), but also meet the requirements for estimating the randomness test of the variables. Therefore, in this paper, in order to analyze the impact of digital finance for homogeneous competition in the banking industry from the network perspective as well as the inter-bank association perspective, this method is used to further research the article topics.

#### 3.3.2. Empirical model

In order to explore the effects of digital finance on the competitive structure of the banks comprehensively and multi-dimensionally, this paper adopts an empirical approach and establishes the models shown in Equations (3, 4) to investigate the relationship between digital finance and banking competition network (banking co-opetition network), where CCNet is the co-opetition network of the explained variable in Equation (3), WCNet is the competition network of the explained variable in the Equation (4), SZ is the explanatory variable which denotes a level of digital financial development., α is the constant term, β and γ are the coefficients of the variables, and μ is the random disturbance term. Meanwhile, this paper refers to Bolt and Humphrey (2010), Tang et al. (2016), Feng and Guo (2019), and Liu and Cuevas (2021), to include control variables (Control) such as earning power (ROA), deposit dependence (DR), business diversification (BD), and debt servicing ability (DPA) in Equations (3, 4). Variables description is shown in Table 1.

(3) $$\begin{array}{l} CCNet=\alpha +\beta_{1}SZ+\beta_{2}BD+\beta_{3}ROA+\\ \;\;\;\;\;\;\;\;\;\;\;\;\;\;\;\;\;\;\;\;\;\beta_{4}DR+\beta_{5}DPA+\mu \end{array}$$

(4) $$\begin{array}{l} WCNet=\alpha +\beta_{1}SZ+\beta_{2}BD+\beta_{3}ROA+\\ \;\;\;\;\;\;\;\;\;\;\;\;\;\;\;\;\;\;\;\;\;\;\beta_{4}DR+\beta_{5}DPA+\mu \end{array}$$

**Table 1:** Variable description.

Variable symbol	Variable name	Indicator
CCNet	Coopetition relationship	Coopetition network
WCNet	Competition relationship	Weighted competition network
Df	Digital financial development	Difference between two banks’ digital financial indices
ROA	Return capacity	Difference between two banks’ return on assets
DR	Deposit dependence	Difference between two banks’ (deposits/total assets)
BD	Business diversification	Difference between two banks’ (non-interest income/total income)
DPA	Solvency	Difference between the shareholders’ equity ratio of two banks

*ROA*, revenue capacity; *DR*, deposit dependence; *BD*, business diversification; *DPA*, debt service capacity.

## 4. Empirical analysis

### 4.1. Sample and data

The sample of this paper is consists of 16 listed banks in China, including Bank of Beijing (BJ), Everbright Bank (GD), industrial and Commercial Bank of China (GS), Huaxia Bank (HX), Construction Bank (JS), Bank of Communications (JT), Minsheng Bank (MS), Bank of Nanjing (NJ), Bank of Ningbo (NB), Agricultural Bank (NY), Ping An Bank (PA), Pudong Development Bank (PF), Industrial Bank (XY), China Merchants Bank (ZS), Bank of China (ZG), and CITIC Bank (ZX). China Merchants Bank (ZS), Bank of China (ZG), and CITIC Bank (ZX). The sample ranges from January 2010 to December 2020. After the global financial crisis broke out in 2007, countries around the world entered a financial recovery period until 2010 when the economy gradually began to recover, and at this time the banking industry in China also ushered in a new turning point and began a new transformation (Xiang, 2010). Meanwhile, the 16 banks cover all State-owned banks, some joint-stock commercial banks, and urban commercial banks in China, with total assets accounting for more than 60% of the total assets of the banking industry. Therefore, the sample is more representative for portraying the competition as well as the development of digital finance.

This paper uses bank-company loan data taken from the CSMAR database. During the data processing, the data are cleaned, screened, and missing values are removed. In order to analyze the heterogeneity of competition structure and digital financial development, subsamples are divided in the data processing. The full samples are divided into two sub-samples. The sub-sample 1 covers all the large banks, including Industrial and Commercial Bank of China (GS), Construction Bank (JS), Bank of Communications (JT), Agricultural Bank (NY), and Bank of China (ZG). the sub-sample 2 involves the small and medium-sized banks, i.e., the remaining 11 listed commercial banks, including Bank of Beijing (BJ)), Everbright Bank (GD), Huaxia Bank (HX), Minsheng Bank (MS), Bank of Nanjing (NJ), Bank of Ningbo (NB), Ping An Bank (PA), Pudong Development Bank (PF), Industrial Bank (XY), China Merchants Bank (ZS), and CITIC Bank (ZX). A total of 70,506 bank-company oberservations were available during the sample period, including a total of 2,871 lending companies.

### 4.2. Analysis of digital finance and banking competition

**Figure 2:**
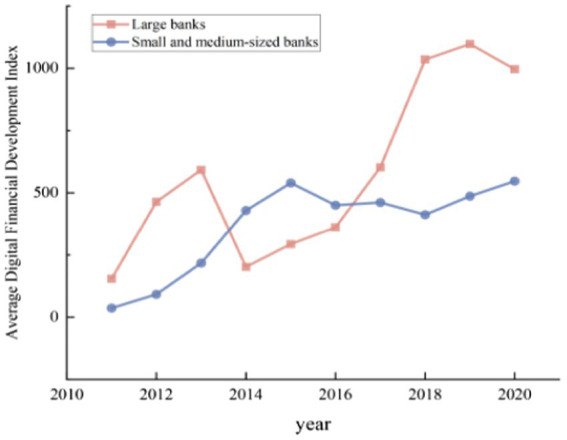
Bank digital finance index.

Figure 2 shows the trends of digital finance of big banks and small and medium sized banks. It is evident that the bank’s digital finance index is on the rise, regardless of big banks or small banks. Moreover, the digital finance index of large banks is generally higher than small and medium-sized banks. Large banks can implement digital transformation more quickly since they have greater capital strength, more developed operational systems and so on.

**Figure 3:**
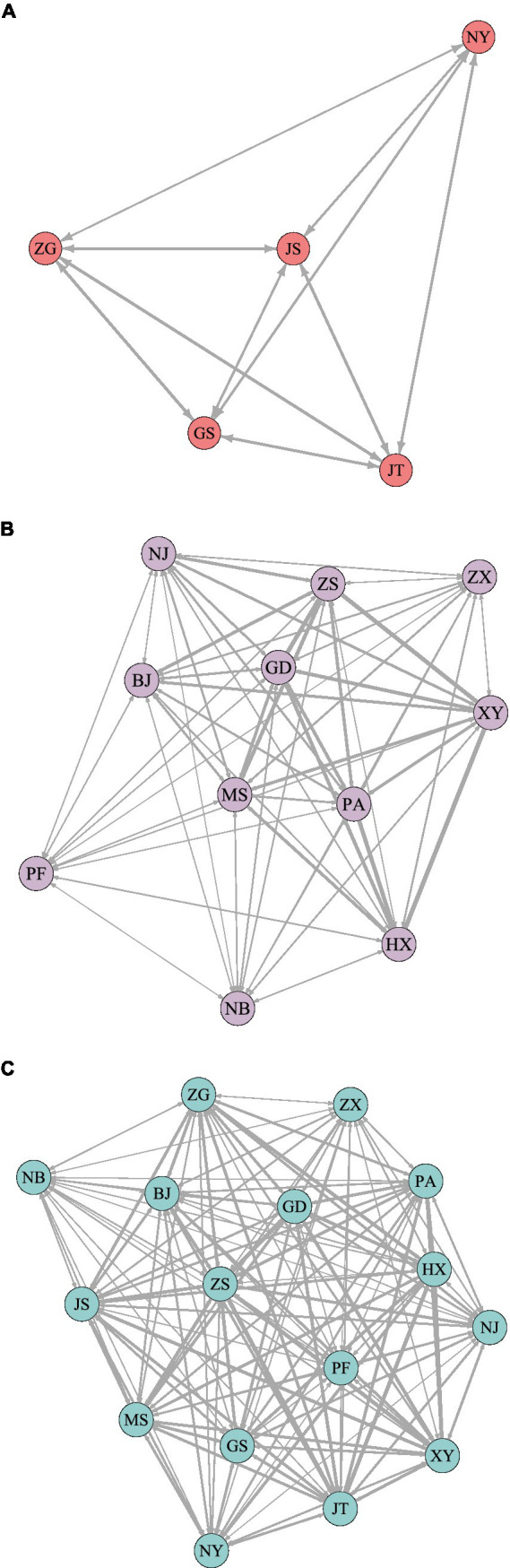
Co-opetition network. BJ, Bank of Beijing; GD, Everbright Bank; GS, Industrial and Commercial Bank; HX, Huaxia Bank; JS, FIGURE 3 (Continued)Construction Bank; JT, Bank of Communications; MS, Minsheng Bank; NJ, Bank of Nanjing; NB, Bank of Ningbo; NY, Agricultural Bank; PA, Ping An Bank; PF, Pudong Development Bank; XY, Industrial Bank; ZS, China Merchants Bank; ZG, Bank of China; and ZX, CITIC Bank, respectively. **(A)** sub-sample network. **(B)** sub-sample2 network. **(C)** full-sample network.

The lending data contains syndication loans and independent loan data of each bank, which can not only effectively characterize inter-bank cooperation, but also can explain the competitive relationship among them. Based on the social network model, this paper uses the number of cooperative loans to characterize the co-competition among banks, and uses the number of joint loans as the weight of the edges in the competition network to construct sub-sample 1 (large banks), sub-sample 2 (small and medium-sized banks), and the full sample competition network, as shown in Figure 3, where the width of the network edge represents the competition weight.

It can be seen from the co-opetition network that it is easier for large state-owned banks to form cooperative relationships with other banks. We can see that the average node strength of the sub-sample 1 (large banks) competitive network in 2020 was 312721.2, and the average node strength of the full sample co-opetition network was 274228.9. That is to say, the average node strength of large banks is significantly higher than the average node strength across the full sample, demonstrating that small and medium-sized banks face less competition than large banks. At the same time, the formation of associated groups, in which large banks cross-aggregate with small and medium-sized banks, rather than a network characterized by the aggregation of large banks with large banks and small banks with small banks, was revealed by the analysis of the characteristics of the cohesive subgroups of the competing networks. For example, Bank of China, Bank of Nanjing, and China Merchants Bank have formed a strongly connected subgroup. This demonstrates that common customers and loans between large and small banks are increasing, as is the similarity of loans between them. The pattern that large banks serve large companies and small banks serve small companies is gradually being changed. Small banks and large banks are increasingly serving the real economy in the form of syndicated loans, which objectively reduces the idiosyncratic risks of each bank, but the increase in loan similarity may exacerbate systemic risks to a certain extent.

**Figure 4:**
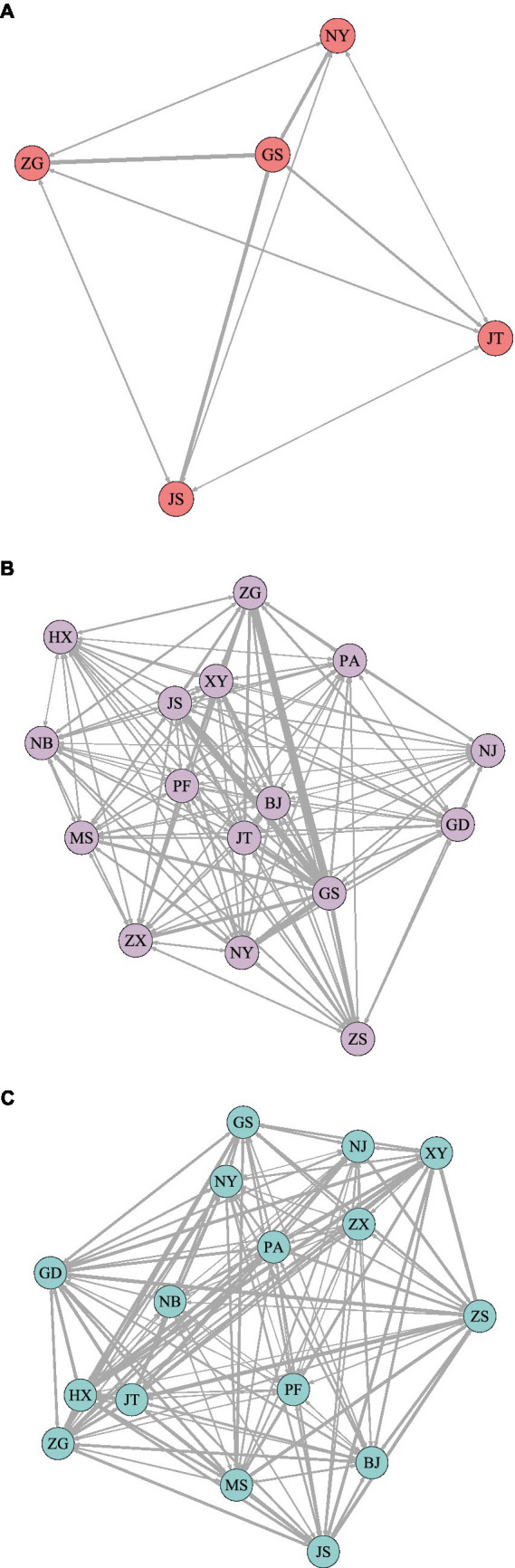
Weighted competitive network. **(A)** Sub-sample 1. **(B)** Sub-sample 2. **(C)** Full sample. BJ, Bank of Beijing; GD, Everbright Bank; FIGURE 4 (Continued)GS, Industrial and Commercial Bank; HX, Huaxia Bank; JS, Construction Bank; JT, Bank of Communications; MS, Minsheng Bank; NJ, Bank of Nanjing; NB, Bank of Ningbo; NY, Agricultural Bank; PA, Ping An Bank; PF, Pudong Development Bank; XY, Industrial Bank; ZS, China Merchants Bank; ZG, Bank of China; and ZX, CITIC Bank, respectively.

**Figure 5:**
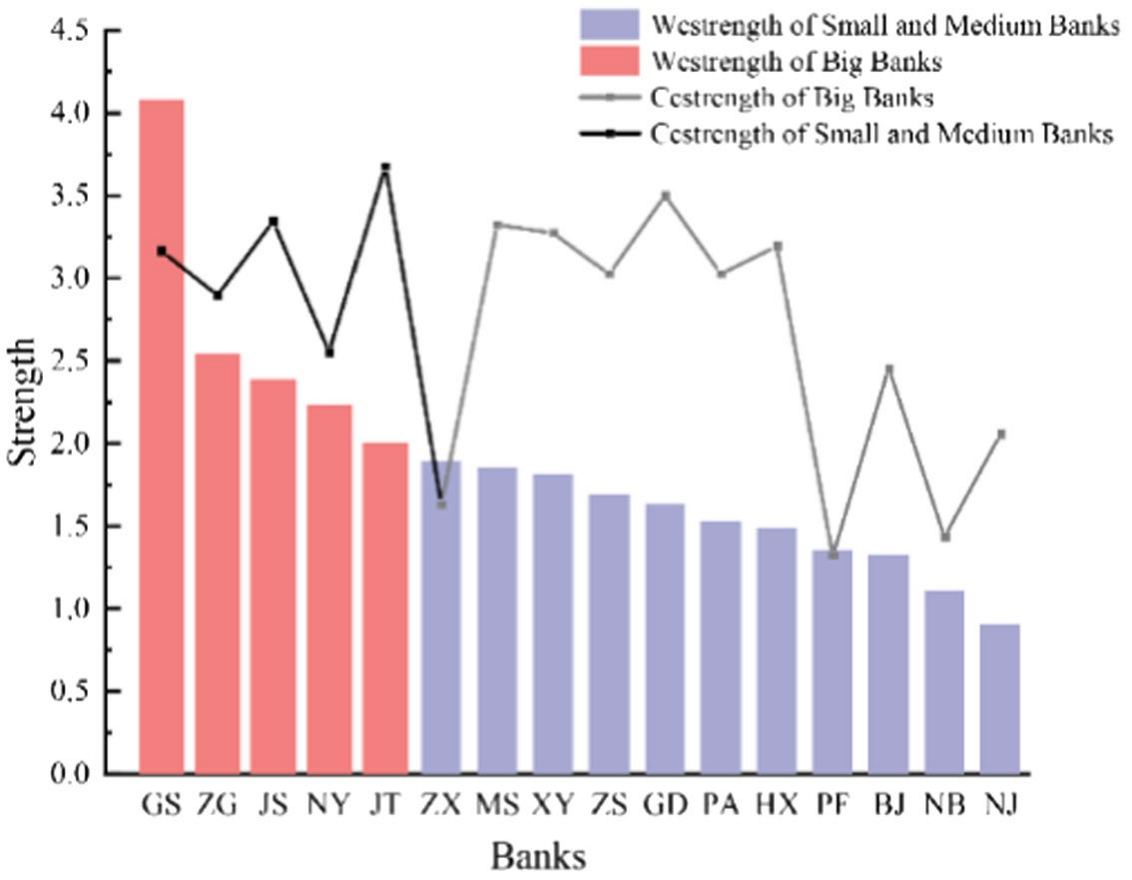
Nodes’ strength of banking networks.

Based on the co-opetition networks, weighted competitive networks are built using one-model credit data and the aforementioned market commonality model, see Figure 4. The market commonality value is utilized in the WNet to describe the competitive pressure that banks exert on one another and is used as the weight of the connected edges of the network. The results of the full-sample network show that large banks’ competitive pressure is significantly stronger than small and medium-sized banks. Furthermore, the average node strength of large banks is 2.65, which is higher than the full sample average node strength of 1.86. Figure 5 shows that the five major state-owned banks’ competitiveness is among the top five.

### 4.3. Correlation analysis

Correlation analysis is able to test whether there is a significant relationship between the variables in the model. Therefore, here correlation tests and analyses were conducted for each variable in model (1) and model (2) above. In the test, the matrices of each variable were replaced 10,000 times in order to ensure the accuracy of the model estimation. The specific test results are shown in Table 2,

**Table 2:** Table of correlation test results.

	*all_CC*	*all_WC*	*all_SZ*	*all_ROA*	*all_DR*	*all_BD*	*all_CC*
*all_DPA*	1	0.36^***^	0.309^**^	0.338^**^	0.214	0.204	0.171
*all_WC*	0.36^***^	1	0.601^***^	0.614^***^	0.488^***^	0.449^**^	0.254^*^
*all_SZ*	0.309^**^	0.601^***^	1	0.634^**^	0.892^***^	0.145	0.173
*all_ROA*	0.338^**^	0.614^***^	0.634^**^	1	0.482^*^	0.409^*^	0.688^***^
*all_DR*	0.214	0.488^***^	0.892^***^	0.482^*^	1	0.085	0.245
*all_BD*	0.204	0.449^**^	0.141	0.409^*^	0.085	1	0.147
*all_DPA*	0.171	0.254^*^	0.173	0.688^***^	0.245	0.147	1

To distinguish with later, the prefix “all_” indicates a full sample of variables. In addition, *CCNet*, denotes coopetition network; *WCNet*, weighted competition network; SZ, digital finance; *ROA*, revenue capacity; *DR*, deposit dependence; *BD*, business diversification; *DPA*, debt service capacity; “***” indicates a significance level of 1%, “**” indicates a significance level of 5%, and “*” indicates significance levels of 10%.

From the results, we can see that the digital finance of banks are significantly correlated with the dependency variables, which is inter-bank competition network and inter-bank competition network, respectively. In addition, banks’ return on assets (ROA), deposit dependence (DR), and business diversification (DB) are significantly correlated with the explanatory variable WNet at 0.5% significance level; banks’ return on assets (ROA) is significantly correlated with interbank co-opetition network. This indicates that the effect of digital finance for banks is closely related to both interbank cooperation and competition, while deposit dependence and business diversification are only closely related to interbank competition.

### 4.4. Empirical test

This paper uses QAP (secondary assignment procedure) to conduct an empirical analysis of the impact of digital finance on banking competition. In order to enhance the accuracy of the model, the matrices of each variable are randomly replaced 10,000 times for estimation. The estimated results of model (3) and model (4) are shown in Table 3, respectively, and their adjusted verdict coefficients are 14.2 and 48.5%, which indicates that the explanatory and control variables in each model could explain 14.2 and 48.5% of the structural changes in the cooperative and competitive relationships. According to the overall regression results, the growth of digital finance has a favorable impact on the competition in the banking sector, and banks’ accelerated adoption of digital transformation and digital finance business helps foster competition among banks, which supports the argument made by academics like Xie and Zou (2012), Wu and Wang (2021), and Bejar et al. (2022). Specifically, model (3) suggests that digital finance, profitability, and business diversification will promote competition among banks. However, the variable of bank deposits has no significant impact on the competitive relationship between banks. This demonstrates that, from the standpoint of network relations, digital finance has increased cooperation opportunities for banks, and to some extent, it can obtain corporate information at a lower cost and create more channels for loan cooperation. This shows that through digital finance, banks can not only promote the enhancement of their competitiveness but also improve the bank’s position in the entire network system. The above analysis illustrates that the impact of digital finance on banks’ competitiveness is quite favorable. The regression estimates of the large bank samples (sub-sample 1), the small and medium-sized bank samples (sub-sample 2), and the bank samples overall (sub-sample 3) are each given below to further clarify the issue and assess the reliability of the aforementioned regression results. See model (5) and model (6) in Table 2 for details, the outcomes resemble those of the full sample estimation. For large-sized banks, digital financial development has no significant impact on inter-bank co-opetition and is only more significantly correlated with banking weighted competitive networks. For small and medium-sized banks, digital finance has a significant impact on both co-opetition and competitive pressure. Which integrate the views of both Dapp (2014), and Bejar et al. (2022).

**Table 3:** Model regression test and robustness test results.

	Model (3)	Model (4)	Model (5)	Model (6)
*all*_*ROA*	0.4340^*^ (1.1749)	0.4950^***^ (0.0000)		
*all_ROA*	0.1923^**^ (4.42392)	0.2201^***^ (0.00223)		
*all_BD*	0.1692^*^ (0.9185)	0.2969^***^ 0.0004		
*all_DR*	−0.2153 (9.3119)	−0.0845 (0.0022)		
*S_SZ*			0.3862^*^ (4.3904)	0.4769^***^ (0.0000)
*S_ROA*			0.1071 (7.2443)	0.1370^*^ (0.0000)
*S_BD*			0.0335 (1.2113)	0.3427^***^ (0.0000)
*R^2^*	0.1709	0.5019	0.1577	0.5425
Adj *R^2^*	0.14203	0.4846	0.1081	0.5156
Number of observations	240	240	110	110
Number of random permutations	10,000	10,000	10,000	10,000

The prefix “all_” is the subsample three variable identifier; The prefix “S” is the subsample 2 variable identifier; SZ, model explanatory variable digital financial development degree; BD, business diversification variable; ROA, return on asset; DR, deposit dependency, DPA, debt-paying ability; “***” indicates a significance level of 1%, “**” indicates a significance level of 5%, and “*” indicates significance levels of 10%.

**Figure 6:**
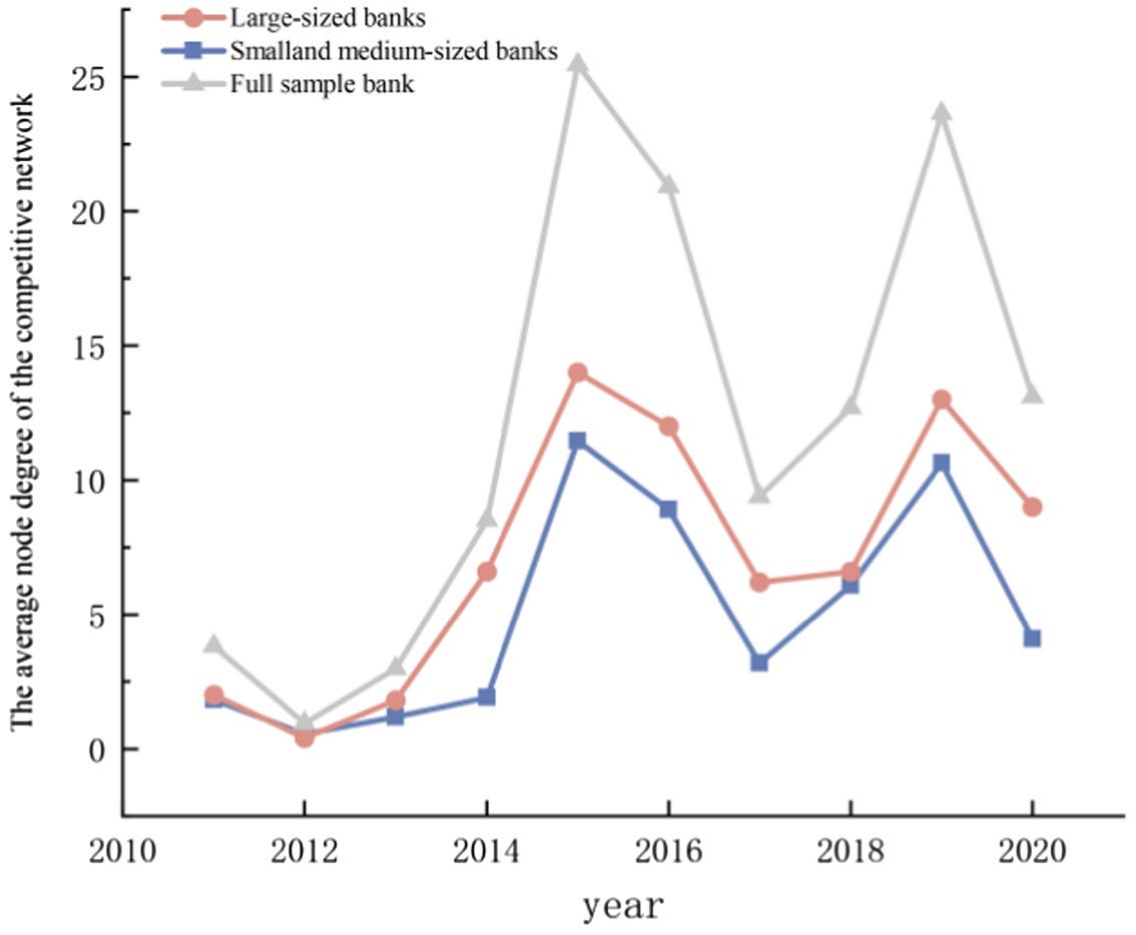
The average node degree of each sample’s competition network.

In order to investigate the similarity of common loans of various types of banks, the degree of nodes was analyzed based on the above-mentioned competitive network (Figure 6), and it was found that the increase of common loans of various types of banks has become a fluctuating upward trend. Combined with the regression results, it can be shown that the development of digital finance has intensified the homogenization competition of small and medium-sized banks to a certain extent.

## 5. Mechanism analysis

The empirical findings show that the growth of digital finance has increased banking competition, and changes in banking competition are bound to affect its operations and business. Therefore, we could trace the evolution of the credit relationship between banks and companies to analyze the mechanism that digital finance affects banking competition. The specific approach is to collect and analyze data on the number of companies to which the 16 banks lend, data on the number of newly added customers of each bank from the other 15 banks, data on the increment of loans in each bank, and data on the average loan of each bank with regards to all its customers during 2010 to 2020.

**Figure 7:**
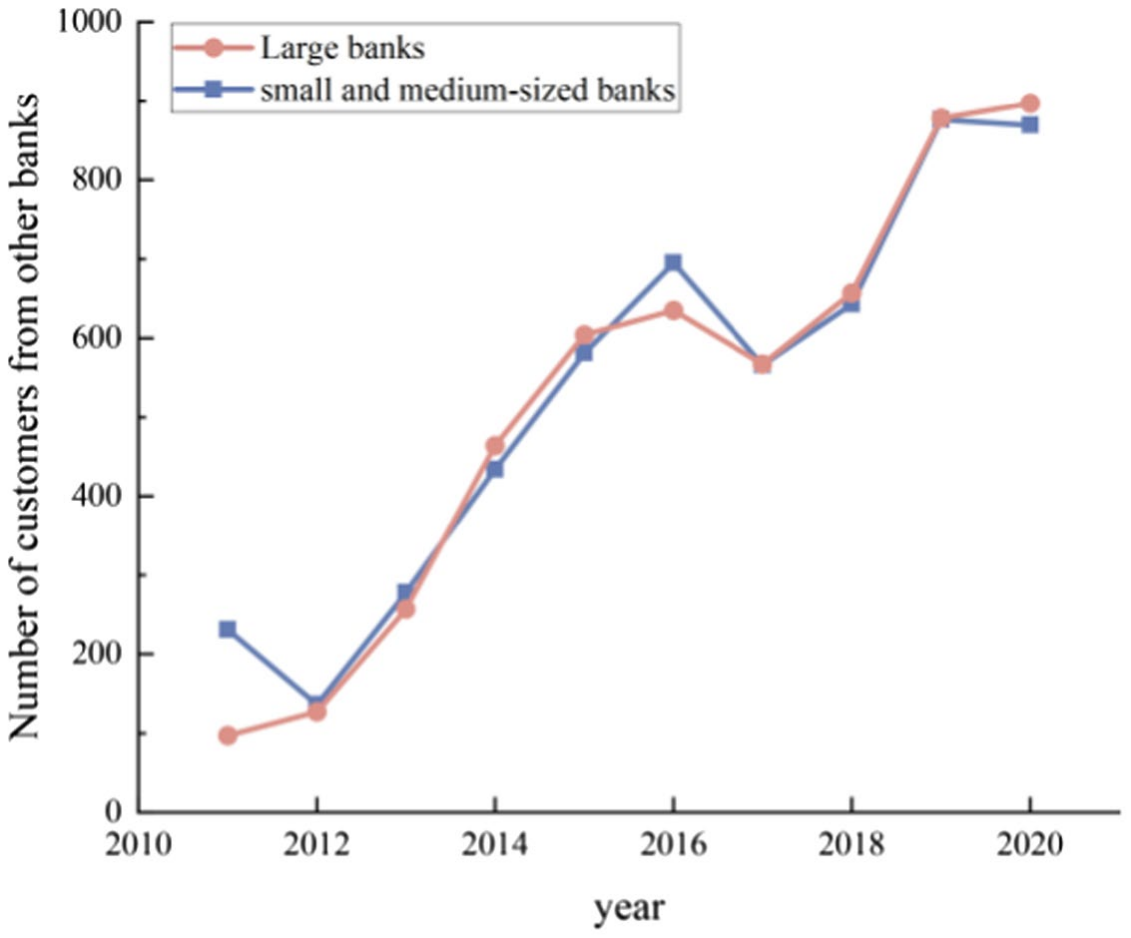
Number of customers from other types of banks among new.

First, the involution of the banking sector has been accelerated by digital finance, which has also objectively pushed optimal allocation of capital. Based on the analysis of new customers of various types of banks, it was found (see Figure 7) that most of new customers in large banks were from the customers of small banks in the last year, while most of new customers in small banks were from the customers of large banks in the last year. Which means that both the large banks and small banks are having more and more joint customers. This finding suggests that the involution of the banking industry has been accelerated by the development of digital finance. The flow or exchange of customers between large banks and small banks objectively shows that the development of digital finance has led to a gradual decline in relational lending, while transactional lending has continued to rise. Digital finance has improved banks’ pricing power and risk identification capabilities, as well as their ability to match supply and demand for funds.

**Figure 8:**
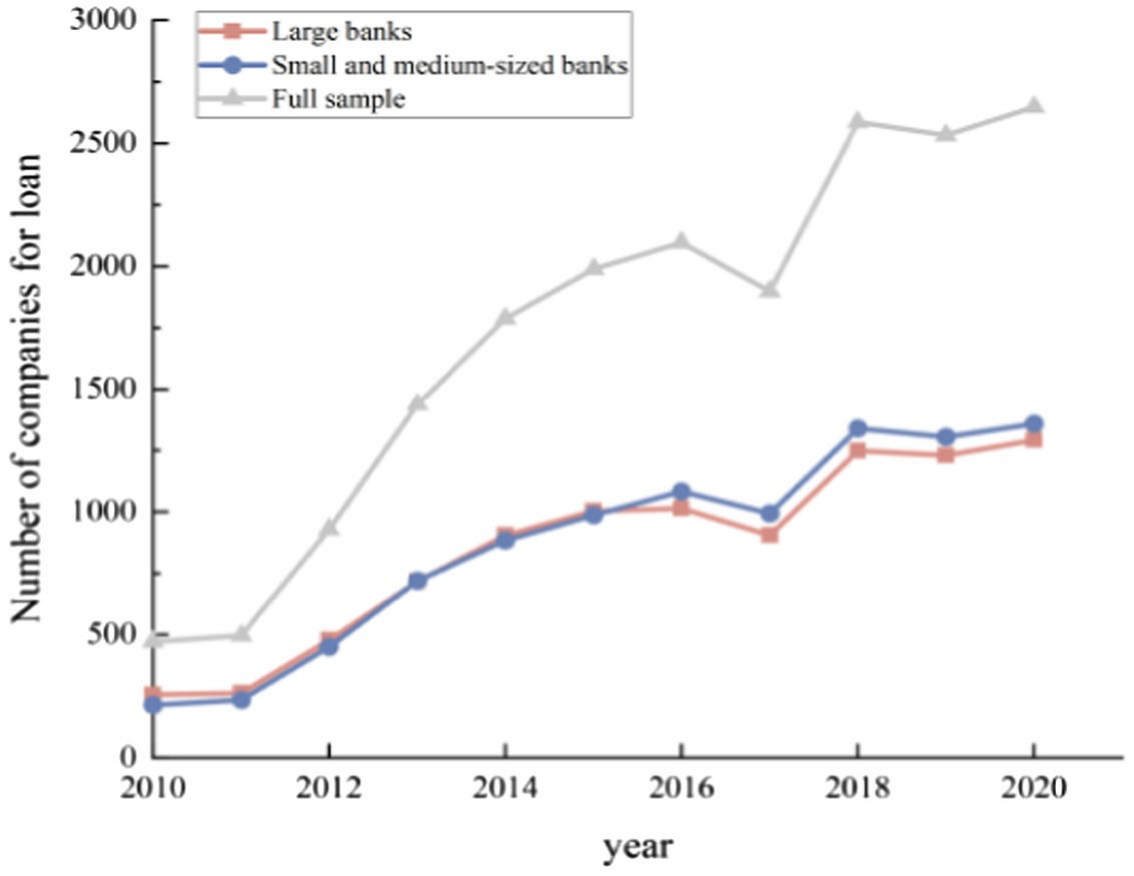
Number of companies for the loan.

Second, digital finance has promoted the evolution of the banking industry, and digital finance has expanded the extension of banking services in the real economy. The number of companies to which the banks provide the loans shows a significant upward trend (see Figure 8). Specifically, the number of companies borrowed from large banks per year, the number of companies borrowed from small and medium-sized banks per year, and the number of companies borrowed from all banks per year take on the same upward trend. This indicates that the increase in the number of large banks’ customers is not entirely from small banks’ customers, and the increase in the number of small banks’ customers is not entirely from large banks’ customers. There is no zero-sum game between large and small banks. Further observation has found that there is a clear co-integration relationship between the number of customers of large banks and number of customers of small banks. It demonstrates that the competition between big banks and small banks has prompted both sides to broaden the width and depth of the services they provide to the real economy, and the expansion of the boundaries of banking services has further boosted the competitiveness and customer acquisition abilities of different types of banks. Digital finance services are effective in the real economy, proving that digital finance has also contributed to the evolution of the banking industry.

**Figure 9:**
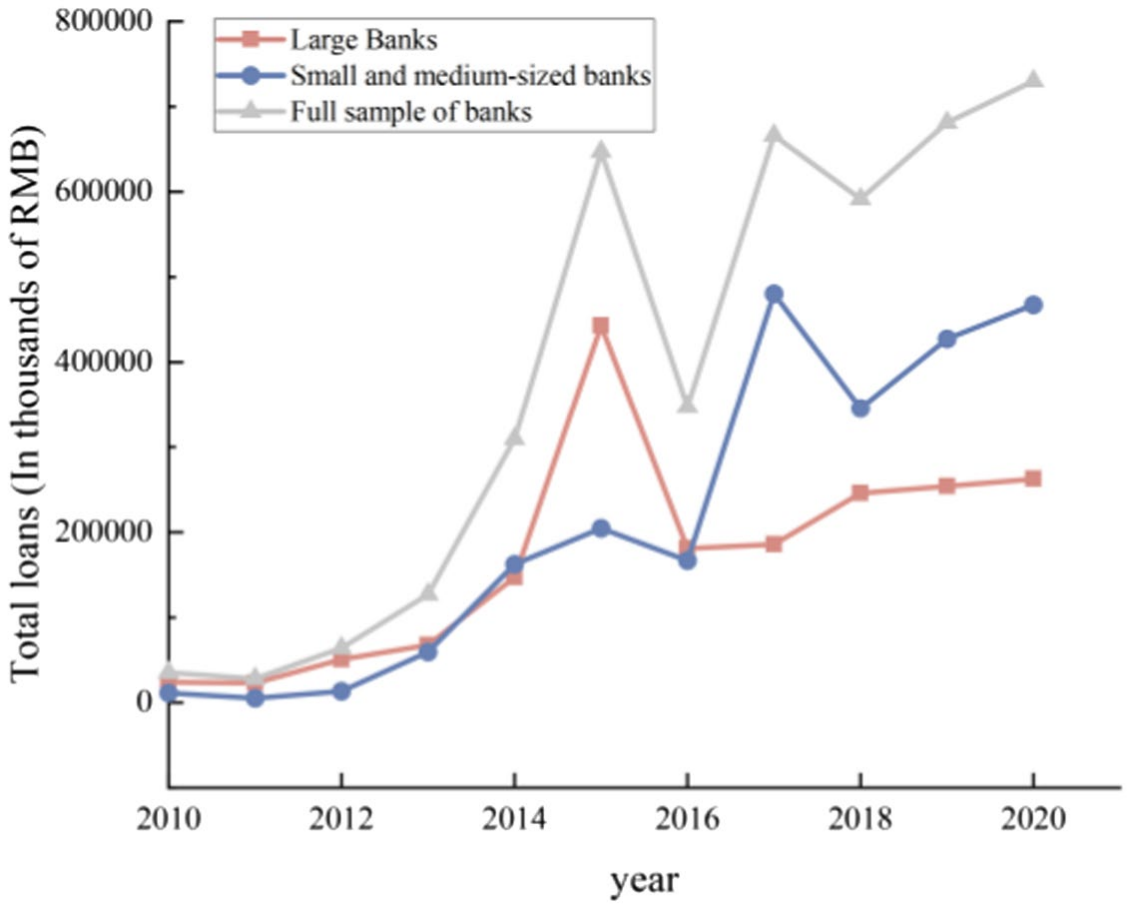
Total loans of banks.

**Figure 10:**
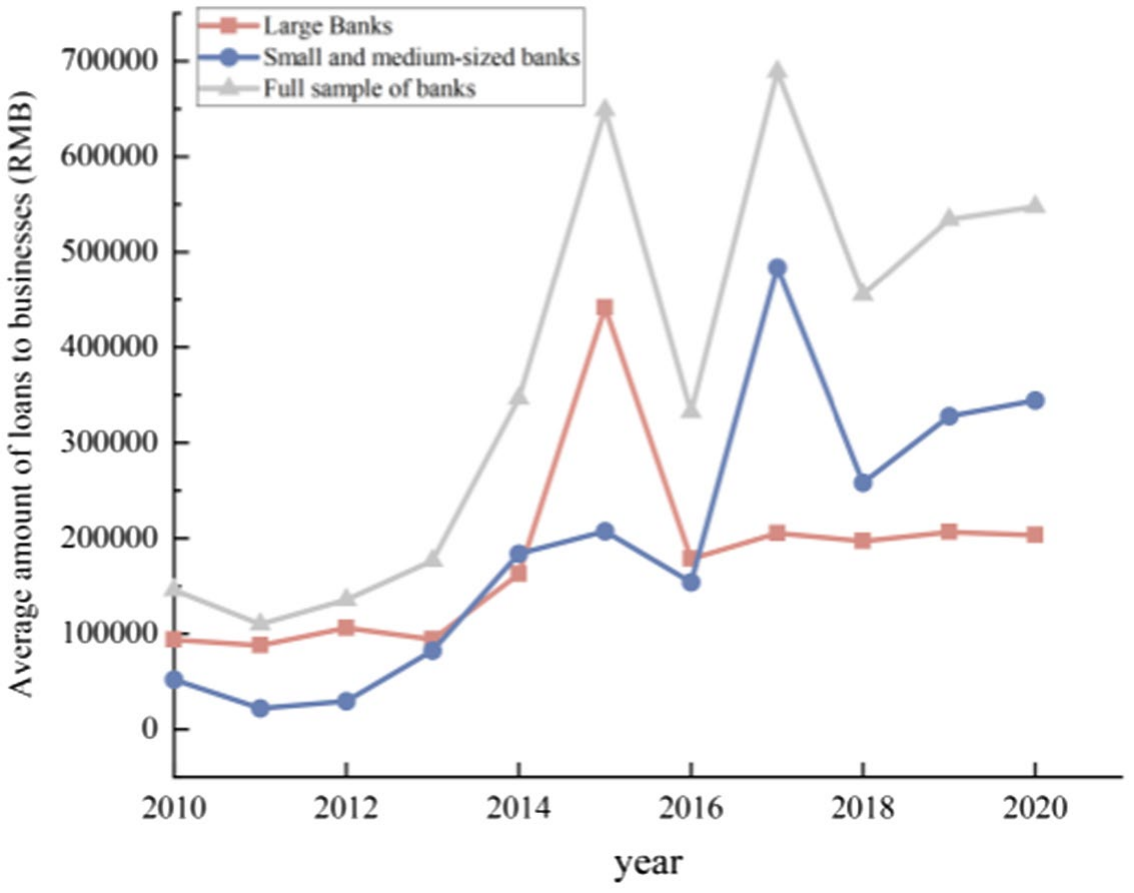
Average amount of bank lending to companies.

Third, digital finance has enhanced the depth of banking services in the real economy. According to the results of total loans from different types of banks (see Figure 9), with the rapid development of digital finance, banks have continuously increased their credit support for the real economy, and the improvement of banking competitiveness has enabled it to meet more credit needs. Meanwhile, according to the statistical results of the average loan amount of various sample banks, it can be found (Figure 10) that the average loan amounts of various types of banks is rising in fluctuation. This demonstrates that driven by digital technology, various types of banks have increased their efforts to serve the real economy while improving their competitiveness. Digital finance has promoted the enhancement of banking service capabilities and is conducive to promoting the real economy to a stage of high-quality development.

In summary, digital finance mainly promotes the overall competitiveness of the banking industry by improving the inclusiveness of financial services, guiding financial services to cover the neglected long-tail parts of the market, and promoting the improvement of the banks’ competitiveness. As a result, the credit structure of the banking industry has been improved and the allocation of credit resources has been optimized, thereby improving the efficiency of the banking and financial business.

## 6. Conclusion and implications

Whether and how digital finance reshapes banking competition structure is a critical issue in modern financial governance. Based on the credit data between banks and companies, this paper used the social network method to build the co-opetition networks based on the common loan data of banks, and build the weighted competition networks based on the market commonality model to measure banking competition. In addition, we used the bank registration and license information to transform the provincial digital financial index into bank-level digital finance. Furthermore, the QAP was used to empirically examine the relationship between the digital finance and banking industry competition.

The study found that digital finance has promoted banking competition, manifesting involution and evolution, which has gradually reduced bank-company relational lending, while transactional lending has continued to rise, banks’ pricing power and risk identification ability have improved, and the ability to optimize the allocation of capital has increased. The competition between large banks and small and medium-sized banks has expanded the scope of serving the real economy. The boundaries of banking services have been expanded, further improving the customer acquisition capabilities and competitiveness of various banks. Therefore, the real economy has benefited greatly from the digital financial services. Digital finance has primarily enhanced the accessibility of financial services, especially for the long-tail customers of the market, improving bank credit structure and optimizing the allocation of credit resources, enhancing banking and financial business efficiency. It is noteworthy that while the growth of digital finance encourages competition, it is critical to be alert of systemic risk that could result from it.

Accordingly, this paper puts forward the following suggestions to improve and optimize the current banking structure. First of all, commercial banks should proactively seize the digital transformation opportunities, taking the service of the real economy as the fundamental, using digital financial empowerment to realize the extension of financial services to large, medium-sized, small or micro companies, and promoting the integration of the real economy and digital finance. Secondly, it is necessary to prevent the occurrence of financial systemic risks caused by excessive homogeneous competition, while avoiding monopolistic behavior caused by excessive unreasonable competition. This requires actively guiding the cooperation among small and medium-sized banks, forcing them to accelerate the application of digital technology and differentiated competition through the form of cooperative innovation of small and medium-sized banks. At the same time, the banking industry and regulators should adhere to the banking franchise and tiered competition to improve the appropriateness of the financial structure to the economic structure. Finally, the state should insist on encouraging the banks and other financial institutions to accelerate the application of digital technology and should strengthen the construction of digital finance, so as to lay the cornerstone for China to enter the stage of high-quality development.

## Data availability statement

Publicly available datasets were analyzed in this study. This data can be found here: https://www.gtadata.com.

## Author contributions

KJ conceptualized the idea and wrote the initial draft of the article. YH performed the analysis. MM validated the results, wrote the methodology section, and proofread the document. All authors contributed to the article and approved the submitted version.

## Conflict of interest

The authors declare that the research was conducted in the absence of any commercial or financial relationships that could be construed as a potential conflict of interest.

## Publisher’s note

All claims expressed in this article are solely those of the authors and do not necessarily represent those of their affiliated organizations, or those of the publisher, the editors and the reviewers. Any product that may be evaluated in this article, or claim that may be made by its manufacturer, is not guaranteed or endorsed by the publisher.
